# Experimental Myocardial Infarction Upregulates Circulating Fibroblast Growth Factor‐23

**DOI:** 10.1002/jbmr.2527

**Published:** 2015-05-06

**Authors:** Olena Andrukhova, Svetlana Slavic, Kathrin I Odörfer, Reinhold G Erben

**Affiliations:** ^1^ University of Veterinary Medicine ViennaViennaAustria

**Keywords:** MYOCARDIAL INFARCTION, FGF23, VITAMIN D

## Abstract

Myocardial infarction (MI) is a major cause of death worldwide. Epidemiological studies have linked vitamin D deficiency to MI incidence. Because fibroblast growth factor‐23 (FGF23) is a master regulator of vitamin D hormone production and has been shown to be associated with cardiac hypertrophy per se, we explored the hypothesis that FGF23 may be a previously unrecognized pathophysiological factor causally linked to progression of cardiac dysfunction post‐MI. Here, we show that circulating intact Fgf23 was profoundly elevated, whereas serum vitamin D hormone levels were suppressed, after induction of experimental MI in rat and mouse models, independent of changes in serum soluble Klotho or serum parathyroid hormone. Both skeletal and cardiac expression of Fgf23 was increased after MI. Although the molecular link between the cardiac lesion and circulating Fgf23 concentrations remains to be identified, our study has uncovered a novel heart–bone–kidney axis that may have important clinical implications and may inaugurate the new field of cardio‐osteology. © 2015 The Authors. *Journal of Bone and Mineral Research* published by Wiley Periodicals, Inc. on behalf of American Society for Bone and Mineral Research (ASBMR).

## Introduction

The prevalence of vitamin D deficiency has been shown to be very high in patients with acute myocardial infarction (MI),^1^ and low serum vitamin D has been implicated in epidemiological studies as a risk factor for hypertension, left ventricular hypertrophy, increased arterial stiffness, and endothelial dysfunction in normal subjects and in patients with chronic kidney disease and type 2 diabetes.^2^, ^3^, ^4^, ^5^, ^6^, ^7^, ^8^ Experimental studies also support a role of vitamin D signaling in the pathophysiology of MI: Vitamin D analogs are cardioprotective in an experimental acute MI model,^9^ and global vitamin D receptor (VDR) knockout mice are characterized by decreased survival and impaired cardiac function post‐MI, relative to wild‐type controls.^9^ However, the mechanisms by which vitamin D may regulate cardiovascular function in MI are still controversial.

Vascular endothelium plays a fundamental role in the regulation of cardiovascular homeostasis through the secretion of vasoactive substances. We recently discovered that vitamin D can regulate endothelial function by modulating the bioavailability of the vasodilator nitric oxide (NO) through transcriptional control of the key NO synthesizing enzyme, endothelial NO synthase (eNOS).^10^ Mice with a nonfunctioning vitamin D receptor (VDR) showed endothelial dysfunction, increased arterial stiffness, increased aortic impedance, structural remodeling of the aorta, and impaired systolic and diastolic heart function.^10^ Endothelial dysfunction is one of the initial events associated with MI and progression of ischemic heart failure (IHF).^11^ In addition, increased arterial stiffness and endothelial dysfunction are related to acute MI incidence and development of chronic heart failure.^12^, ^13^ Therefore, we hypothesized that reduced vitamin D levels in MI might contribute to endothelial dysfunction observed post‐MI. Indeed, use of vitamin D additives in the normal population has been linked to lower MI incidence.^14^ On the other hand, vitamin D supplementation in patients with a history of MI did not improve endothelial function or other markers of vascular function.^15^ In addition to its role for the regulation of vascular tone, vitamin D may also directly influence cardiomyocyte function because mice with a cardiomyocyte‐specific deletion of the vitamin D receptor develop cardiac hypertrophy.^16^

One of the most potent suppressors of vitamin D hormone production is fibroblast growth factor 23 (FGF23), a bone‐derived hormone secreted by osteoblasts and osteocytes in response to increased circulating vitamin D hormone and phosphate levels.^17^, ^18^ FGF23 signals through a receptor complex consisting of FGF receptor‐1c and the transmembrane protein α‐Klotho (Klotho).^19^ FGF23 increases renal phosphate excretion by inhibiting renal tubular phosphate reuptake through downregulation of the apical membrane abundance of sodium‐phosphate cotransporters in renal proximal tubules.^17^, ^20^, ^21^, ^22^ At the same time, FGF23 suppresses the transcription of renal 1α‐hydroxylase in renal proximal tubules, the key enzyme in the vitamin D activation pathway.^17^ The crucial role of Fgf23 in the control of renal 1α‐hydroxylase is illustrated in Fgf23‐ and Klotho‐ablated mice, suffering from early mortality because of unleashed 1α‐hydroxylase activity and the toxic effects of severe hypervitaminosis D.^23^, ^24^

In addition to its phosphaturic and vitamin D hormone‐suppressive actions, FGF23 may have an important role for cardiovascular health. Several large epidemiological studies have linked increased circulating FGF23 with left ventricular mass and hypertrophy, impaired left ventricular function, vascular dysfunction, and increased risk for MI.^25^, ^26^, ^27^, ^28^ In addition, it is well established that circulating FGF23 is positively and dose‐dependently associated with cardiovascular risk factors such as left ventricular hypertrophy, vascular calcifications, and mortality in patients with chronic kidney disease.^29^, ^30^ Moreover, circulating FGF23 levels have been shown to independently predict clinical outcome in patients with established heart failure.^31^ The molecular mechanisms by which FGF23 may influence cardiovascular function are still controversial. Recently, we found that FGF23 directly regulates membrane abundance and activity of the Na^+^Cl^‐^ cotransporter NCC in distal renal tubules and increases renal sodium reabsorption and plasma volume.^32^ This finding suggests that FGF23 is not only a phosphaturic but also a sodium‐conserving hormone involved in volume and blood pressure homeostasis. Therefore, excessive FGF23 signaling may induce cardiac hypertrophy by salt and volume retention, leading to hypertension and increased afterload. In addition, FGF23 may contribute to left ventricular hypertrophy by a direct, Klotho‐independent action on cardiomyocytes.^30^

The two abovementioned lines of evidence led us to hypothesize that FGF23 might be involved in the pathogenesis of cardiac dysfunction after MI. To test our hypothesis, we induced experimental MI in two different rat and mouse animal models by permanent or transient ligation of the left descending coronary artery. Here, we show that experimental MI results in a striking upregulation of circulating intact Fgf23 together with suppressed vitamin D hormone levels in both rat and mouse models, uncovering a novel endocrine link between heart, bone, and kidney.

## Materials and Methods

### Animals

All animal procedures were approved by the Ethical Committee of the University of Veterinary Medicine Vienna. Male 16‐week‐old Fischer 344 rats or C57BL/6 mice were kept under standardized conditions in groups of 2 to 5 animals at 24°C and a 12‐hour light/dark cycle with free access to tap water and a normal rodent diet. At necropsy, the rats and mice were exsanguinated from the abdominal aorta and the abdominal V. cava, respectively, under anesthesia with ketamine/xylazine (67/7 mg/kg ip) for serum collection.

### Mouse acute myocardial infarction model

Mouse acute myocardial infarction model MI was induced in mice by permanent ligation of the left anterior descending coronary artery. Briefly, left lateral thoracotomy was performed and a suture was tightened around the left anterior descending coronary artery under anesthesia with ketamine/medetomidine (100/0.25 mg/kg ip) mice and controlled ventilation with room air. Sham‐operated animals underwent the same surgical procedure with the exception of coronary ligature. Pain was managed by buprenorphine treatment. All mice were killed 4 weeks after MI or sham surgery.

### Rat ischemia‐reperfusion model

Ischemia‐reperfusion was induced in rats as previously established.^33^ Briefly, rats underwent surgery under general anesthesia by i.p. injection of medetomidine/fentanyl/midazolam (150 μg/kg/5 μg/kg/2 mg/kg). Ischemia/reperfusion injury was induced by ligating the left descendent coronary artery for 30 min followed by reperfusion under controlled ventilation with 100% oxygen. Anesthesia was antagonized with atipamezol/flumazenil/naloxone (0.75/0.2/0.12 mg/kg) at the end of surgery. Pain was managed by metamizol and carprofen treatment. Sham‐operated animals underwent the same surgical procedure with the exception of coronary ligature. Rats were killed 2 and 4 weeks after MI or sham surgery.

### Transthoracic Doppler echocardiography

Transthoracic Doppler echocardiography was performed 3 weeks after MI under 1.5% to 2% isofluorane anesthesia using a 14 MHz linear‐array transducer (Acuson s2000tm, Siemens,). LV dimensions in systole and diastole were measured at the level of papillary muscles, and fractional shortening (FS) was calculated. A minimum of four cardiac cycles were averaged for each parameter.

### Biochemical analyses

Serum calcium, sodium, phosphorus, and alkaline phosphatase activity, as well as urinary creatinine, calcium, sodium, and phosphorus were analyzed using a Cobas c111 analyzer (Roche, Mannheim, Germany). Serum intact Fgf23 (Kainos, Tokyo, Japan), 1,25(OH)_2_D (IDS, Gaithersburg, MD, USA), PTH (Immutopics, San Clemente, CA, USA), OPG (Immundiagnostik, Bensheim, Germany), and Dkk‐1 (rat: Enzo Life Sciences, Farmingdale, NY, USA; mouse: R&D Systems, Minneapolis, MN, USA) were determined using commercially available ELISAs. Total deoxypyridinoline (DPD) concentrations in urine were determined after acid hydrolysis by ELISA (total DPD, Quidel, San Diego, CA, USA).

### Western blot

Heart tissue and aorta homogenates or serum samples were mixed with Laemmli sample buffer, fractionated on SDS‐PAGE (50 μg/well), and transferred to a nitrocellulose membrane (Thermo Fisher Scientific, Waltham, MA, USA). Immunoblots were incubated overnight at 4°C with primary antibodies, including monoclonal rat anti‐FGF23 (1:2000, kindly provided by Amgen Inc., Thousand Oaks, CA, USA), polyclonal rabbit anti‐αKlotho (1:2500, Abcam, Cambridge, MA, USA; membrane‐bound and shed forms), polyclonal rabbit anti‐eNOS (1:2000, Novus Biologicals, Littleton, CO, USA), polyclonal anti‐albumin (Abcam), monoclonal mouse anti‐GAPDH (1:500, Millipore, Billerica, MA, USA), and monoclonal mouse anti‐β‐actin (1:5000, Sigma, St. Louis, MO, USA) in 2% (w/v) bovine serum albumin (BSA, Sigma) in a TBS‐T buffer (150 mM NaCl, 10 mM Tris [pH 7.4/HCl], 0.2% [v/v] Tween‐20). After washing, membranes were incubated with horseradish peroxidase‐conjugated secondary antibodies (Amersham Biosciences, Piscataway, NJ, USA). Specific signal was visualized by ECL kit (Amersham Life Sciences). The protein bands were quantified by Image Quant 5.0. Each experiment included 4 to 8 animals per group.

### Histology and immunohistochemistry

Hearts were fixed in 4% paraformaldehyde (PFA), embedded in paraffin, and sectioned at 5‐µm thickness. Cardiac fibrotic tissue was visualized by Masson's trichrome staining. Before immunohistochemical staining, dewaxed sections were pretreated for 60 minutes with blocking solution containing 5% normal goat serum in PBS with 0.1% bovine serum albumin and 0.3% Triton X‐100. Endogenous peroxidases were blocked by pretreatment with hydrogen peroxide before incubation with primary antibody. Sections were incubated with monoclonal rat anti‐FGF23 (kindly provided by Amgen Inc.) antibodies at 4°C overnight. After washing, sections were incubated for 1.5 hours with peroxidase‐labeled rabbit anti‐rat (Invitrogen, Carlsbad, CA, USA, 1:400) secondary antibody with subsequent diaminobenzidine (DAB, Sigma) staining. Immunostaining of tissue sections in which primary antibodies were omitted served as a negative control. The slides were analyzed on a Zeiss Axioskop 2 (Carl Zeiss Microscopy, Thornwood, NY, USA) microscope.

### Quantification of aortic calcium and phosphate content

Aortas were homogenized and the calcium and phosphate content was determined in the supernatant by using commercially available kits (Greiner Diagnostic, Bahlingen, Germany). Calcium and phosphate contents were normalized to the wet weight of the aorta.

### Statistical analyses

Statistics were computed using SPSS for Windows 17.0 (SPSS, Inc., Chicago, IL, USA). The data were analyzed by two‐sided *t* test (2 groups) or one‐way analysis of variance (ANOVA) followed by Student‐Newman‐Keuls multiple comparison test (>2 groups). Any *p* values less than 0.05 were considered significant. The data are presented as the mean ± SEM.

## Results

**Figure 1:**
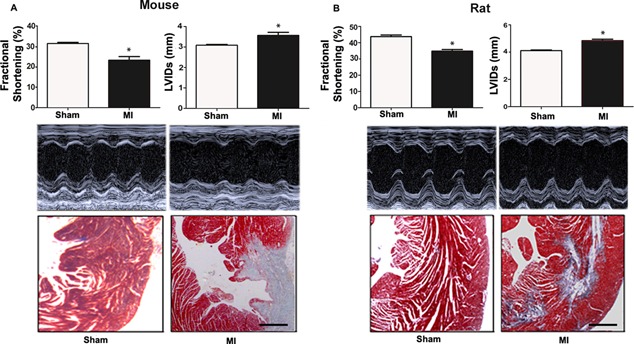
Cardiac function is impaired after MI. Fractioning shortening and left ventricular systolic internal diameter (LVIDs) were measured by echocardiography (ECG) in mouse (*A*) and rat (*B*) experimental MI models 3 weeks post‐MI or sham surgery. MI was induced in mice by permanent ligation of the left descending coronary artery and by ischemia‐reperfusion in rats. Middle panels show original ECG images. Bottom panels show blue‐stained fibrotic tissue in left ventricles of MI animals by Masson trichrome staining of paraffin sections 4 weeks post‐MI. Data represent mean ± SEM of 6 to 7 animals per group. *p < 0.05 versus Sham. Scale bar = 100 μm.

As expected, induction of experimental MI resulted in fibrotic left ventricular lesions and impaired cardiac function, both in mice after permanent ligation of the left descending coronary artery and in rats after ischemia‐reperfusion (I‐R) injury with transient ligation of the left coronary artery (Fig. 1). Echocardiography showed similar impairments in fractional shortening and similar increases in systolic left ventricular internal diameter in both experimental MI models, 3 weeks after MI (Fig. 1). We included the rat I‐R model in the present study because this model closely resembles the injury induced by myocardial infarction and primary reperfusion therapies in humans and because it increases the breadth of the study with the inclusion of two different species.

**Figure 2:**
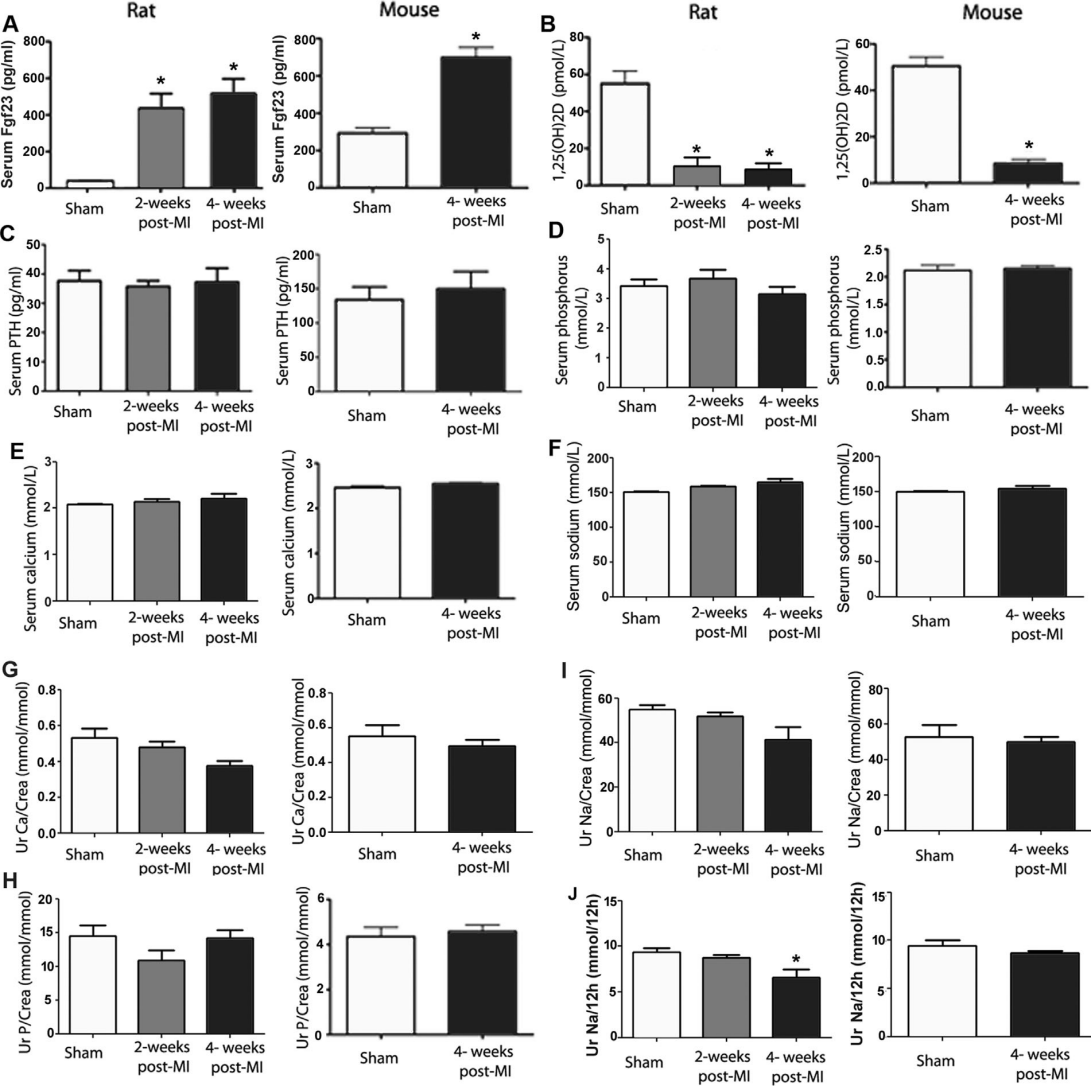
Circulating Fgf23 is increased and serum vitamin D hormone is suppressed in mouse and rat MI models. Serum Fgf23 (*A*), serum vitamin D hormone (*B*), serum PTH levels (*C*), serum phosphorus (*D*), serum calcium (*E*), serum sodium (*H*), urinary phosphate/creatinine ratio (*F*), urinary calcium/creatinine ratio (*G*), urinary sodium/creatinine ratio (*I*), and urinary sodium excretion per 12 hours (*J*) in MI‐ and sham‐operated mice (at 4 weeks postsurgery) and rats (at 2 and 4 weeks postsurgery). Data represent mean ± SEM of 6 to 7 animals in each group for A–I and of 5 to 6 animals in each group for J. *p < 0.05 versus Sham.

To validate our hypothesis regarding the involvement of FGF23 signaling in the pathophysiology of cardiac dysfunction after MI, we measured circulating FGF23 levels in both animal models. We found striking increases in serum Fgf23 in mice and rats after experimental MI (Fig. 2 *A*). Although this was actually our hypothesis, we were surprised by the magnitude of changes in circulating intact Fgf23 in MI animals. Four weeks after MI, serum Fgf23 was about 10‐fold higher in MI rats and about 2.5‐fold higher in MI mice relative to sham‐operated controls. Conversely, serum vitamin D hormone levels were markedly suppressed by about 80% in both MI mice and rats compared with sham controls, 4 weeks post‐MI (Fig. 2 *B*). The increase in serum Fgf23 and the suppression of serum vitamin D levels were already evident at 2 weeks post‐MI in rats (Fig. 2 *A, B*). Serum PTH (Fig. 2 *C*), serum phosphate (Fig. 2 *D*), serum calcium (Fig. 2 *E*), serum sodium (Fig. 2 *F*), as well as urinary calcium/creatinine (Fig. 2 *G*), phosphate/creatinine (Fig. 2 *H*), and sodium/creatinine (Fig. 2 *I*) excretion remained unchanged in MI animals. However, in line with our recent finding that Fgf23 is a sodium‐conserving hormone,^32^ we found decreased urinary sodium excretion per 12 hours in MI rats 4 weeks postsurgery (Fig. 2 *J*).

**Figure 3:**
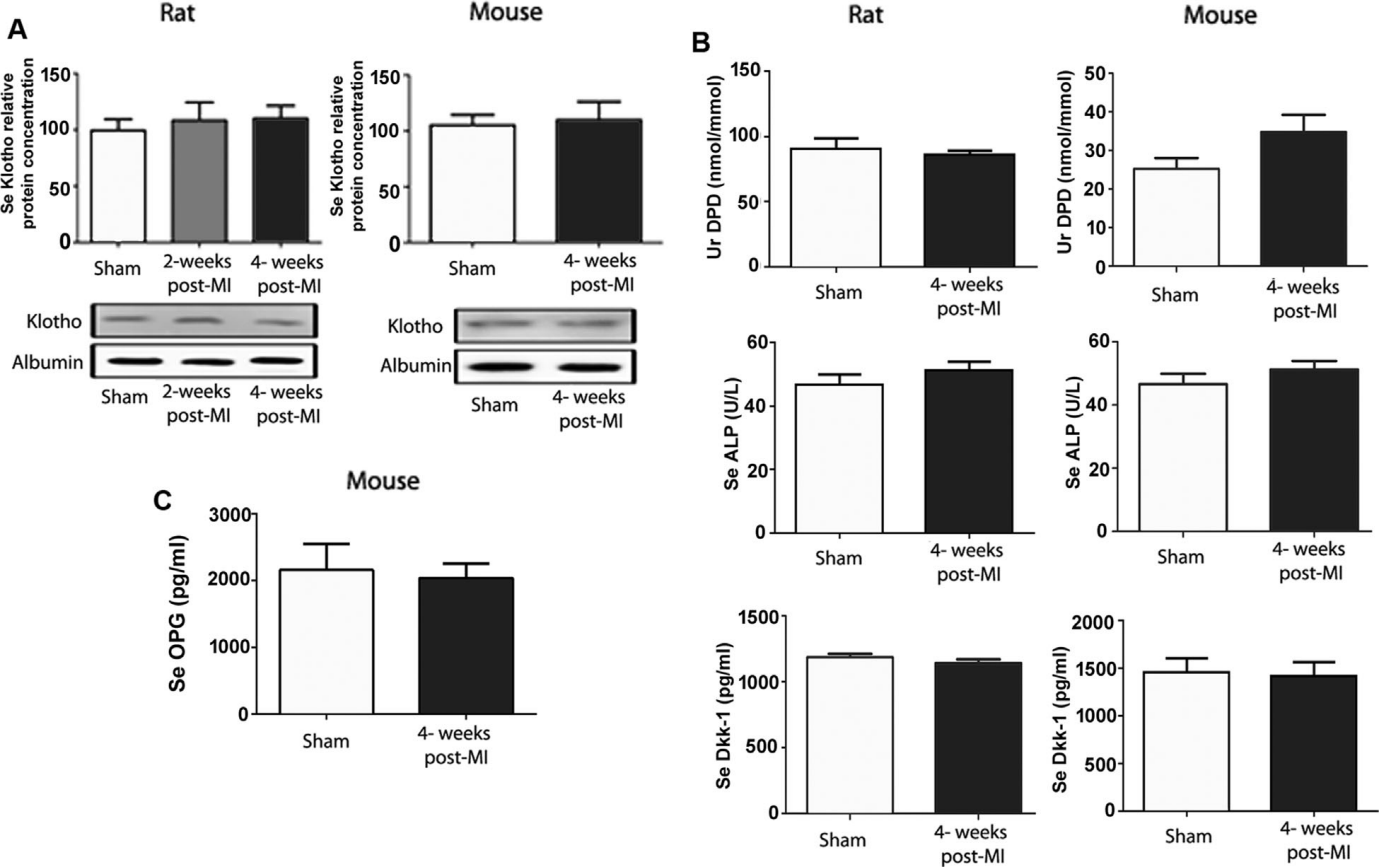
Serum Klotho abundance and biochemical markers of bone metabolism are unchanged after MI. (*A*) Relative serum Klotho protein abundance in mice (at 4 weeks postsurgery) and rats (at 2 and 4 weeks postsurgery). (*B*) Urinary deoxypyridinoline (DPD)/creatinine excretion, serum (Se) alkaline phosphatase (ALP), and serum Dickkopf‐1 (Dkk‐1) in MI‐ and sham‐operated rats and mice 4 weeks post‐MI. (*C*) Serum osteoprotegerin (OPG) in MI‐ and sham‐operated mice 4 weeks post‐MI. Data represent mean ± SEM of 5 to 7 animals in each group.

It has been recently reported that soluble Klotho may be cardioprotective by regulating TRPC6 channels in cardiomyocytes.^34^ Therefore, we measured the relative abundance of soluble Klotho in serum by Western blotting. However, serum Klotho remained unchanged in MI rats and mice (Fig. 3 *A*). Because Fgf23 is a bone‐derived hormone, we assessed bone metabolism by measuring urinary excretion of the collagen cross‐link deoxypyridinoline, together with serum alkaline phosphatase, Dickkopf‐1, and osteoprotegerin (the latter measured only in mice). However, we found no differences between MI rats and mice relative to Sham controls in any of these biochemical markers of bone metabolism 4 weeks post‐MI (Fig. 3 *B, C*).

**Figure 4:**
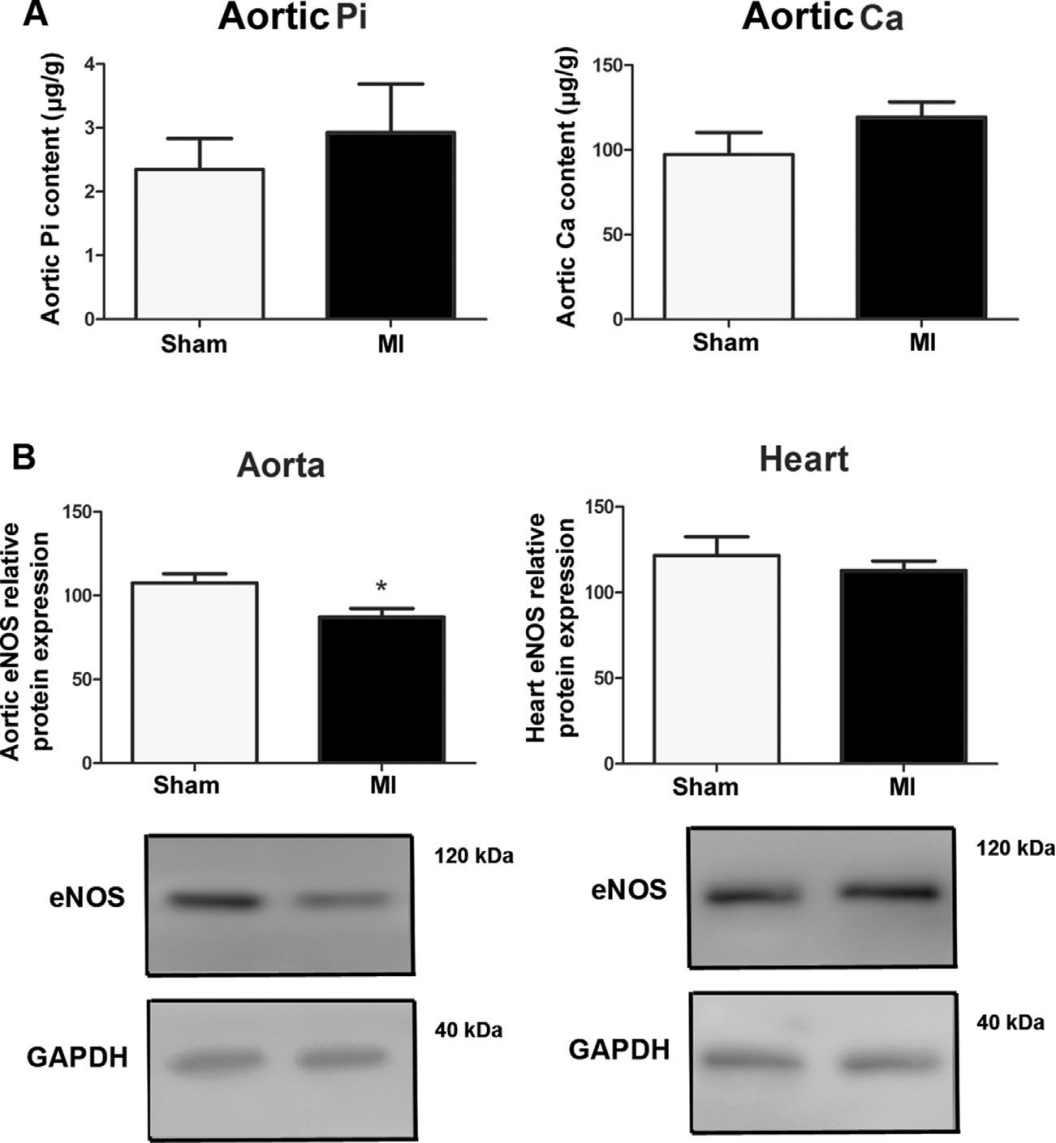
Aortic calcification is unchanged, but aortic eNOS protein expression is reduced after MI. Aortic inorganic phosphate (Pi) and calcium (Ca) content (*A*) and Western blotting analysis of eNOS protein expression in aorta and heart of MI and sham‐operated mice (*B*) 4 weeks post‐MI. Data represent mean ± SEM of 4 to 6 animals in each group. *p < 0.05 versus Sham.

To examine vascular calcification after MI, we measured calcium and phosphate content in aortic homogenates of Sham and MI rats. We found no evidence of aortic calcification 4 weeks post‐MI (Fig. 4 *A*). We previously reported that the vitamin D hormone is a transcriptional regulator of eNOS in vascular endothelium.^10^ To analyze whether the suppressed vitamin D hormone levels found in MI animals in this study translate into lower vascular or cardiac eNOS protein abundance, we assessed eNOS protein expression by Western blotting. We found unchanged cardiac but significantly reduced aortic eNOS protein expression in MI mice compared with Sham controls (Fig. 4 *B*).

**Figure 5:**
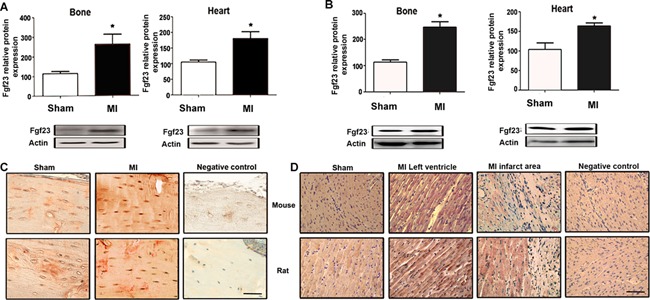
Bone and cardiomyocyte Fgf23 protein expression is increased after MI. Western blotting analysis of femur and heart Fgf23 protein expression in MI‐ and sham‐operated mice (*A*) and rats (*B*) 4 weeks post‐MI. Immunohistological anti‐Fgf23 staining in femurs (*C*) and hearts (*D*) of Sham and MI mice and rats 4 weeks post‐MI. Data represent mean ± SEM of 4 to 6 animals each. *p < 0.05 versus Sham. Scale bar = 20 µm.

Although the major site of FGF23 production is bone, low levels of FGF23 mRNA can also be detected in the heart.^35^ To examine the origin of the increased serum Fgf23 post‐MI, we performed Western blotting analysis of extracts from bones and hearts after MI. Fig. 5 *A, B* show that Fgf23 protein expression was profoundly upregulated in bone post‐MI but also, albeit to a lesser extent, in infarcted hearts. Immunohistological staining revealed increased expression of Fgf23 in osteocytes (Fig. 5 *C*) and left ventricular cardiomyocytes (Fig. 5 *D*) in both MI rats and mice. Infiltrating leukocytes in the infarct scar were Fgf23‐negative (Fig. 5 *D*). The latter findings suggest that viable cardiomyocytes are the major cellular source of increased cardiac Fgf23expression after MI.

## Discussion

**Figure 6:**
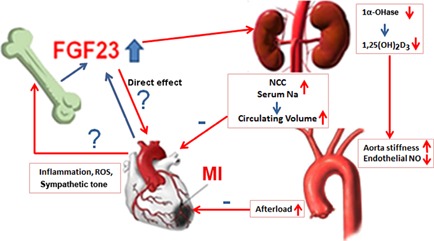
Role of Fgf23 in the pathophysiology of cardiac dysfunction after MI. MI increases serum intact Fgf23 possibly by inflammatory signals, ROS, or increased sympathetic tone. In addition, increased cardiac Fgf23 expression might also contribute to elevated serum Fgf23 post‐MI. Increased circulating intact Fgf23, in turn, stimulates renal NCC expression leading to salt and volume retention, suppresses renal vitamin D hormone production leading to endothelial dysfunction, and may have direct pro‐hypertrophic actions on the heart.

To the best of our knowledge, this is the first study showing that experimental MI augments skeletal and cardiac Fgf23 expression and profoundly upregulates serum concentrations of intact Fgf23. In addition, our data demonstrate that circulating vitamin D hormone concentrations are suppressed in MI rats and mice. Hence, increased Fgf23 could be a major factor contributing to disease progression post‐MI by 1) volume overload through stimulation of renal NCC expression; 2) induction of endothelial dysfunction through suppression of vitamin D hormone production; and 3) possibly direct pro‐hypertrophic actions on the heart. A graphical scheme depicting this vicious circle is shown in Fig. 6. This novel heart–bone–kidney axis may have major clinical implications for the management of patients after MI and for cardiovascular medicine in general. In line with the proposed model, we found reduced aortic eNOS protein expression in MI mice in the current study.

It has been suggested that the FGF23 co‐receptor Klotho may have an FGF23‐independent role in the regulation of cardiac functions, protecting the heart from left ventricular hypertrophy and systolic dysfunction through regulation of TRPC6 channels in cardiomyocytes.^34^ However, in contrast to circulating FGF23, serum soluble Klotho was not found to be associated with left ventricular function or hypertrophy in human cardiology patients.^36^ Similarly, serum soluble Klotho remained unchanged in our murine and rat MI models. Collectively, these data suggest that changes in serum soluble Klotho concentrations have only a minor, if any, role in MI pathophysiology.

The phosphaturic action of Fgf23 has been very well documented, and excessive circulating intact FGF23 actually leads to hypophosphatemia and rickets or osteomalacia.^20^, ^21^ Therefore, it is currently a conundrum why profoundly elevated serum levels of intact Fgf23 post‐MI do not translate into hypophosphatemia and hyperphosphaturia in the presence of unchanged serum PTH, the other major phosphaturic hormone. We don't have a conclusive answer to this question. However, it is possible that lower cardiac output post‐MI might interfere with the phosphaturic action of FGF23 by lowering the amount of filtered phosphate.

Animal experimental studies have shown that vitamin D, NCC inhibition, and overexpression of eNOS all have beneficial effects on cardiovascular function post‐MI. A recent study in VDR‐deficient mice showed that lack of VDR signaling has a detrimental effect on survival post‐MI and that administration of a vitamin D analogue partially protects against the deleterious cardiovascular effects of experimental MI.^9^ In addition, administration of the NCC inhibitor chlorothiazide improves heart function in an MI‐induced model of congestive heart failure.^37^ Global eNOS knockout mice, an ultimate model of endothelial dysfunction, develop concentric left ventricular hypertrophy and fibrosis,^38^, ^39^ indicating the importance of the autocrine and paracrine effects of NO in cardiac remodeling. Along the same line, cardiomyocyte‐specific overexpression of eNOS improves left ventricular performance and reduces compensatory hypertrophy after MI.^40^ Therefore, the protective role of vitamin D, eNOS and NCC inhibitors in MI models underscores the possible importance of Fgf23 as a direct or indirect master regulator of all three factors. Although it is generally thought that eNOS in cardiomyocytes has an overall cardioprotective role, it has to be mentioned in this context that uncoupling of NOS as a consequence of MI can result in the generation of reactive oxygen species instead of NO, resulting in increased oxidative stress and additional cardiac damage.^41^

The crucial question why circulating Fgf23 is increased after experimental MI is currently unclear. It is known that hypervitaminosis D, hyperphosphatemia, and increased circulating PTH stimulate Fgf23 secretion in bone.^18^ However, serum PTH and phosphate remained unchanged, and serum vitamin D hormone concentrations were actually suppressed in our experiments, suggesting that other factors must be responsible for the increase in serum FGF23 post‐MI. In addition, biochemical markers of bone metabolism were not increased after MI in the current study, making it unlikely that changes in bone metabolism account for the changes in Fgf23 expression after MI. Under physiological circumstances, Fgf23 secretion from osteocytes and osteoblasts is believed to be the major source of circulating intact Fgf23.^42^ Because we observed an upregulation of both osteocytic and cardiomyocyte Fgf23 expression post‐MI, it is currently unclear whether cardiac Fgf23 secretion contributes to the rise in circulating Fgf23 post‐MI. It is possible that pro‐inflammatory cytokines and ROS, which are elevated post‐MI,^43^ could be the link between MI and increased osteocytic Fgf23 expression. It is interesting to note in this context that circulating FGf23 levels have been found to correlate with pro‐inflammatory markers in CKD patients.^44^ Therefore, pro‐inflammatory cytokines originating from the cardiac lesion may stimulate skeletal Fgf23 secretion post‐MI. Because it was recently reported that sympathetic activation may upregulate skeletal Fgf23 secretion,^45^ it is also conceivable that increased sympathetic tone may stimulate osteocytic Fgf23 secretion after MI. Clearly, more work has to be performed to characterize the molecular mechanisms linking heart and bony Fgf23 secretion.

In conclusion, our study has uncovered that experimental MI results in profoundly upregulated levels of intact circulating Fgf23. Elevated serum levels of FGF23 might induce a vicious circle after MI, putting additional strain on the heart by salt and volume retention and by endothelial dysfunction caused by suppression of serum vitamin D hormone production. Our findings provide novel insights into the pathophysiology of cardiac dysfunction after MI and may be of importance for the development of new therapeutic strategies for the clinical management of MI patients.

## Disclosures

All authors state that they have no conflicts of interest.
